# A New ERAP2/Iso3 Isoform Expression Is Triggered by Different Microbial Stimuli in Human Cells. Could It Play a Role in the Modulation of SARS-CoV-2 Infection?

**DOI:** 10.3390/cells9091951

**Published:** 2020-08-24

**Authors:** Irma Saulle, Claudia Vanetti, Sara Goglia, Chiara Vicentini, Enrico Tombetti, Micaela Garziano, Mario Clerici, Mara Biasin

**Affiliations:** 1 Department of Biomedical and Clinical Sciences-L. Sacco, University of Milan, 20157 Milan, Italy; irma.saulle@unimi.it (I.S.); claudia.vanetti@unimi.it (C.V.); sarag9623@gmail.com (S.G.); chiara.vicentini@studenti.unimi.it (C.V.); enrico.tombetti@unimi.it (E.T.); micaela.garziano@unimi.it (M.G.); 2 Department of Pathophysiology and Transplantation, University of Milan, 20122 Milan, Italy; mario.clerici@unimi.it; 3 Don C. Gnocchi Foundation ONLUS, IRCCS, 20148 Milan, Italy

**Keywords:** ERAP2, ERAP2/Iso3, microbial infections, alternative splicing, SARS-CoV-2, host cell response

## Abstract

Following influenza infection, rs2248374-G ERAP2 expressing cells may transcribe an alternative spliced isoform: ERAP2/Iso3. This variant, unlike ERAP2-wt, is unable to trim peptides to be loaded on MHC class I molecules, but it can still dimerize with both ERAP2-wt and ERAP1-wt, thus contributing to profiling an alternative cellular immune-peptidome. In order to verify if the expression of ERAP2/Iso3 may be induced by other pathogens, PBMCs and MDMs isolated from 20 healthy subjects were stimulated with flu, LPS, CMV, HIV-AT-2, SARS-CoV-2 antigens to analyze its mRNA and protein expression. In parallel, Calu3 cell lines and PBMCs were in vitro infected with growing doses of SARS-CoV-2 (0.5, 5, 1000 MOI) and HIV-1_BAL_ (0.1, 1, and 10 ng p24 HIV-1_Bal_/1 × 10^6^ PBMCs) viruses, respectively. Results showed that: (1) ERAP2/Iso3 mRNA expression can be prompted by many pathogens and it is coupled with the modulation of several determinants (cytokines, interferon-stimulated genes, activation/inhibition markers, antigen-presentation elements) orchestrating the anti-microbial immune response (Quantigene); (2) ERAP2/Iso3 mRNA is translated into a protein (western blot); (3) ERAP2/Iso3 mRNA expression is sensitive to SARS-CoV-2 and HIV-1 concentration. Considering the key role played by ERAPs in antigen processing and presentation, it is conceivable that these enzymes may be potential targets and modulators of the pathogenicity of infectious diseases and further analyses are needed to define the role played by the different isoforms.

## 1. Introduction

ERAP1 and ERAP2 (endoplasmic reticulum aminopeptidases 1 and 2) are two IFNγ- and TNFα-inducible, ubiquitously-expressed human enzymes, which belong to the M1 family of zinc aminopeptidases [1]. In the endoplasmic reticulum (ER), ERAPs shape the antigenic repertoire by trimming the N-terminus of precursor peptides previously generated in the cytoplasm by the proteasome. In this way, ERAPs generate optimal-length peptides for loading onto MHC class I groove to be presented to CD8+ T lymphocytes [2,3]. Despite maintaining marked differences in their enzymatic specificity these two enzymes can act together in a concerted way, through the formation of homo- or heterodimers, thus allowing the generation of a variegated and more immunogenic antigenic repertoire [4]. In particular, ERAP1–ERAP2 heterodimer generation has been demonstrated to improve the shaping of peptides suitable for MHC class I molecule binding [5].

ERAPs are encoded by two genes, sharing ~49% sequence homology and situated on chromosome 5q15 in opposite directions, which are highly polymorphic [6]. Since their leading role in the antigen processing pathway, several studies have investigated any potential link between ERAP polymorphic variants and alterations in their functioning, which could result in MHC-I-associated disorder onset [2,7,8] as well as into variations in susceptibility/progression to microbial infections [9].

**Figure 1:**
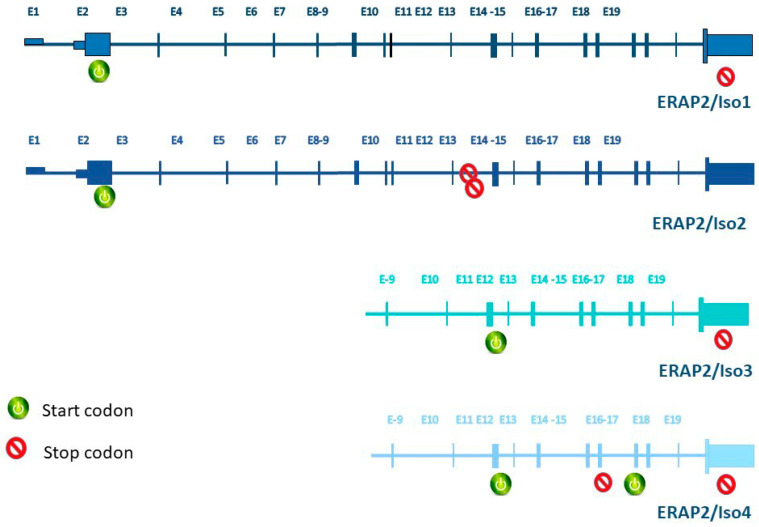
Genetics of ERAP2 isoforms regulation. Structures of transcripts derived from each ERAP2 isoform are represented. Start and stop codons for each isoform are reported.

As for ERAP2, the most relevant single nucleotide polymorphism (SNP) is the non-coding rs2248374 (A/G) which identifies two haplotypes, hereafter referred to as HapA (A allele for rs2248374) and HapB (G allele for rs2248374). In HapB, the G allele for this SNP primes the transcription of a spliced ERAP2 variant (ERAP2/Iso2), presenting an extended exon 10 (56 extra nucleotides) and two in-frame TAG stop codons, which in turn lead to its nonsense-mediated decay (NMD) [10]. Conversely, HapA is translated into a 965-amino-acid protein and is associated with Crohn’s disease [11], HLA-A29-associated birdshot uveitis [12], ankylosing spondylitis [13,14] and juvenile idiopathic arthritis [15], as well as natural resistance to HIV infection [9,16,17]. Since these variants are maintained by a balanced selection to a frequency of approximately 50% (HapB: 53% and HapA: 47%), nearly 25% of the population fails to express the ERAP2 protein [18]. This observation raises a logic question: in which peculiar setting does balancing selection operate to conserve the apparently loss-of-function HapB and the disease-causing HapA in the human population? Quite recently, Ye and co-workers provided an exhaustive explanation to this apparent paradox [19]. Indeed, for the first time, they documented the transcription of two novel short isoforms (ERAP2/Iso3, ERAP2/Iso4) from flu-infected monocyte-derived dendritic cells isolated from homozygous HapB-carrying subjects [19]. The two short isoforms are transcribed from HapB and differ from the full-length one—ERAP2/Iso1, transcribed from HapA—since their transcription begins in correspondence of exon 9 and undergoes alternative splicing of an extended exon 10. Besides, they diverge from each other by alternative splicing at a secondary splice site at exon 15 (Figure 1). Of note, while ERAP2/Iso4 is predicted to harbor a premature termination codon that could lead to NMD, ERAP2/Iso3, is expected to be translated into a protein [19]. Such protein misses the catalytic domain [19], but could still critically contribute to profile the cellular immune-peptidome as it preserves the capacity to dimerize with ERAP1 and possibly ERAP2-wild type (wt) [20].

Based on these premises, the aim of our study was to investigate if the transcription of ERAP2/Iso3 is either flu-specific or if it can be induced by other kind of stimuli, such as other viruses, bacteria, or inflammatory triggers. Given the foremost role played by ERAPs in the field of both acquired and innate immunity, the characterization of the different isoforms produced in a particular pathological setting, such as the one caused by microbial infections, could lead to the identification of new molecular targets to be exploited in the setting up of innovative therapeutically or vaccinal approaches.

## 2. Methods

### 2.1. Study Population

Twenty healthy controls (HC) were enrolled in the study in order to investigate the induction of ERAP isoform transcription in response to common recall antigens such as influenza-antigens (flu), Lipopolisaccaride (LPS), Citomegalovirus (CMV), in addition to Aldrithiol-2 (AT-2)-inactivated R5-tropic human immunodeficiency virus-1_Ba-L_ (HIV-AT-2) and acute respiratory syndrome coronavirus 2 (SARS-CoV-2) inactivated virus (i-SARS-CoV-2). The Ethical Committee of the Fondazione IRCCS Ca’ Granda Ospedale Maggiore Policlinico approved the study (Prot. N°0028257). All the donors signed an informed consent form, in agreement with the Declaration of Helsinki of 1975, revised in 2013.

### 2.2. Viruses

The laboratory-adapted HIV-1 strain used in the experiments was the R5 tropic HIV-1_BaL_ (courtesy of Drs. S. Gartner, M. Popovic, and R. Gallo; NIH AIDS Research and Reference Reagent Program) provided through the EU program EVA Centre for AIDS Reagents (NIBSC, Potter Bars, UK). The virus was inactivated with AT-2, which is able to change the zinc finger nucleocapsid proteins of HIV-1, therefore deactivating the viral infectivity as previously described [21].

SARS-CoV-2 (Virus Human 2019-nCoV strain 2019-nCoV/Italy-INMI1, Rome, Italy) was expanded on Calu-3 cells (ATCC^®^ HTB-55™) and TCID_50_ was calculated as previously reported [22]. SARS-CoV-2 inactivation (i-SARS-CoV-2) was obtained by incubation at 65 °C for 30 min [23].

### 2.3. ERAP2 Genotyping Analyses 

Whole blood was collected by all the subjects enrolled in the study by venipuncture in Vacutainer tubes containing EDTA (BD Vacutainer, San Diego, CA, USA). Total DNA was extracted by DNA purification Maxwell^®^ RSC Instrument (Promega, Fitchburg, WI, USA) and quantified using the Nanodrop 2000 Instrument (Thermo Scientific, Waltham, MA, USA). Two-hundred ng of DNA were used to perform an SNP genotyping assay for ERAP2 rs2549782 (G/T) (TaqMan SNP Genotyping Assay; Applied Biosystems, Foster City, CA, USA), which is in linkage disequilibrium with the non-coding rs2248374 (A/G). Analyses were performed on Peripheral blood mononuclear cells (PBMCs) from all the subjects recruited in the study as well as on lung adenocarcinoma cells (Calu3; ATCC^®^ HTB-55™). Allelic discrimination real-time PCR method was used to analyze the results.

### 2.4. Isolation of PBMCs and Monocyte-Derived Macrophages (MDMs) Differentiation

PBMCs, obtained from density gradient centrifugation on Ficoll (Cedarlane Laboratories Limited, Hornby, ON, Canada), were counted by automated cell counter ADAM-MC (NanoEnTek Inc., Seoul, Korea), which distinguishes viable from non-viable cells.

Flow cytometer analyses was used to quantify the percentage of CD14+ monocytes in PBMCs isolated from 3 HeteroAB HC. MDMs were obtained as previously described [24]. Briefly, 5 × 10^5^ adherent monocytes were incubated for 5 days in RPMI with 20% of fetal bovine serum (FBS) (Euroclone, Milan, Italy) and 100 ng/mL macrophage-colony stimulating factor (M-CSF) (R&D Systems, Minneapolis, MN, USA). Optical microscope (ZOE™ Fluorescent Cell Imager, Bio-Rad, Hercules, CA, USA) observation allows to verify MDM differentiation.

### 2.5. Cell Cultures for Microbial Antigen Stimulation

PBMCs were resuspended at the concentration of 1 × 10^6^ PBMCs/mL in RPMI 1640 medium (Euroclone, Milan, Italy) containing 10% fetal bovine serum (FBS), 1% levo-glutammin LG and 2% penstreptomicin. Subsequently, PBMCs and MDMs from HC were stimulated with antigens from different pathogens: 32 µg/mL of CMV grade 2 Antigen (Microbix Biosystem, Mississauga, ON, Canada), 1 µg/mL of LPS, 1 ng/mL of HIV-AT-2 equivalents and 5 multiplicity of infection (MOI) of i-SARS-CoV-2 inactivated virus. Two live UV-inactivated influenza viruses (flu) were used as well: an influenza A virus (A/RX73 and A/Puerto Rico/8/34 strains; 1:800) and the 1998–1999 formula of flu vaccine (1:5000; Wyeth Laboratories Inc., Marietta, PA, USA). Cells were stimulated even with non-microbial stimuli: 100U of IFNα and 1 µg/mL of IL-1β (Sigma, Saint Louis, MO, USA). Unstimulated PBMCs were cultured as control as well. Cells were harvested 10 (PBMCs) and 36 (MDMs) h post-treatment for RNA and protein analyses, respectively.

### 2.6. In Vitro Infection of PBMCs and Calu3 Cells with SARS-CoV-2

2.5 × 10^5^ Calu3 cells (ATCC^®^ HTB-55™) were cultured in DMEM medium (Euroclone, Milan, Italy) supplemented with 2% FBS in a 24-well plate. DMEM containing 100 U/mL penicillin and 100 μg/mL streptomycin was used as inoculum in the mock-infected cells. Cell cultures were incubated with serial dilutions of virus supernatant in duplicate, (1000 MOI, 5 MOI, 0.5 MOI) for three h at 37 °C and 5% CO_2_. Cells were washed two times with lukewarm PBS and refilled with the proper growth medium (10%FBS). Optical microscope observation (ZOE™ Fluorescent Cell Imager, Bio-Rad, Hercules, CA, USA) was performed daily to investigate the cytopathic effect. The infected cells were harvested for SARS-CoV-2 detection in the supernatant and mRNA collection at 48 h. Each culture condition was run in triplicate. All the procedures were performed in agreement with the GLP guidelines adopted in our laboratory.

Maxwell^®^ RSC Viral Total Nucleic Acid Purification Kit (Promega, Fitchburg, WI, USA) was used to extract RNA from Calu3 cell culture supernatants by the Maxwell^®^ RSC Instrument (Promega, Fitchburg, WI, USA). Viral RNA was quantified, by single-step RT PCR -time PCR (GoTaq^®^ 1-Step RT-qPCR) (Promega, Fitchburg, WI, USA) on a CFX96 (Bio-Rad, Hercules, CA, USA) by using TaqMan probes which target two portions of SARS-CoV-2 nucleocapsid (N) gene (N1 and N2). Specifically, we used the 2019-nCoV CDC qPCR Probe Assay emergency kit (IDT, Coralville, IA, USA), which allows also to amplify the human RNase P gene. Viral copy number quantification was performed by generating a standard curve from the quantified 2019-nCoV_N positive Plasmid Control (IDT, Coralville, IA, USA). 

### 2.7. In Vitro HIV-Infection Assay

3 × 10^6^ PBMCs from all the subjects included in the study were in vitro HIV-infected as previously described [25] with 10, 1, and 0.1 ng p24 HIV-1_Bal_/1 × 10^6^ PBMCs. After 5 days, supernatants were collected for p24 antigen ELISA (Cell Biolabs, San Diego, CA, USA), whereas PBMCs collected at 2 days post-infection were used for RNA extraction and gene expression analyses.

### 2.8. Gene Expression Analysis

RNA extracted from 1 × 10^6^ PBMCs, and Calu3 cell lines were retrotranscribed as previously described [16]. cDNA quantification for ERAPs was performed on antigen-stimulated and HIV-infected PBMCs as well as on SARS-CoV-2 infected Calu3 cells through a real-time PCR (CFX96 connect, Bio-Rad, Hercules, CA, USA) and an SYBR Green PCR mix (Bio-Rad, Hercules, CA, USA); all the reactions were carried out in duplicate. Results are shown as the media of the relative expression units to the glyceraldehyde-3-phosphate dehydrogenase (GAPDH) and β-actin housekeeping genes calculated by the 2^−ΔΔCt^ equation. The following thermal protocol was used: initial denaturation (95 °C, 15 min) followed by 40 cycles of 15 s at 95 °C (denaturation), 20 s at 60 °C (annealing) and 20 s at 72 °C (extension). Furthermore, a melting curve analysis was assessed for amplicon characterization. Ct values of 35 or higher were let off the analyses.

### 2.9. Quantigene Plex Gene Expression Assay

Gene expression of 8 × 10^5^ PBMCs was performed by quantiGene Plex assay (Thermo Scientific, Waltham, MA, USA) which provides a fast and high-throughput solution for multiplexed gene expression quantitation, allowing the simultaneous measurement of 70 custom selected genes of interest in a single well of a 96-well plate. The QuantiGene Plex assay is hybridization-based and incorporates branched DNA (bDNA) technology, which uses signal amplification for direct measurement of RNA transcripts. The assay does not require RNA purification.

### 2.10. Western Blot Analyses

Cultured MDMs were removed by non-enzymatic cell dissociation solution (Sigma, Saint Louis, Missouri, USA), counted by the automated cell counter ADAM-MC (Digital Bio) and used for protein extraction by RIPA buffer (Sigma, Saint Louis, MO, USA). Extracted proteins were stored at −80 °C for further analyses. For WB analyses, samples from 3 HeteroAB subjects were sub-pooled (50 μg per pool). Equal amounts of proteins were separated by 4–20% SDS-polyacrylamide gel electrophoresis (Criterion TGX Stain-free precast gels and Criterion Cell system; Bio-Rad) and transferred onto nitrocellulose membrane using a Bio-Rad Trans-Blot Turbo System. Membranes were probed using a 1:1000 dilution of primary antibody goat anti-ERAP1 (AF2334; R&D Systems, Minneapolis, MN, USA) goat anti-ERAP2 polyclonal antibody (AF3830; R&D Systems, Minneapolis, MN, USA), rabbit anti-GAPDH polyclonal (VPA00187); Bio-Rad, Hercules, CA, USA] and a 1:10,000 dilution of secondary antibody conjugated with alkaline phosphatase anti-goat IgG (A4187; Sigma; goat anti-rabbit (STAR208P); Bio-Rad]. Membranes were incubated with the appropriate antibody and, after being excited using the Clarity Western ECL substrate, bands were visualized with a ChemiDoc MP imaging system (Bio-Rad) and quantified for densitometry with the Bio-Rad Image Lab software.

### 2.11. Statistical Analyses

Data are shown as mean and standard deviation. Analysis and figures were performed by GRAPHPAD PRISM version 5 (Graphpad software, La Jolla, CA, USA) and SPSS Statistics, version 25 (IBM software, Armonk, NY, USA). Gene expressions of ERAP2/iso1, ERAP2/iso1, and ERAP1 in PBMCs, monocyte-derived macrophages, and Calu-3 cells upon stimulation were compared to that of untreated cells by Wilcoxon test and Mann–Whitney test, as appropriate. P-values were corrected for false discovery rate (FDR) by using Microsoft R software, *p*-values ≤ 0.005 were considered to be significant.

## 3. Results

### 3.1. ERAP2 Allelic Variants Analyses

Analysis of ERAP2 SNP prevalence was aligned with European population distribution reported in the U.S. National Library of Medicine Database [https://www.ncbi.nlm.nih.gov/snp/rs2549782?fbclid=IwAR1ZdwC747PDWvtAzt6hZBV5j7oFZiPkLjY-JdSee1Plzvym7fhJVQc1Aks (data not shown). Among the 20 genotyped HC: 6 were HomoA, 8 heterozygous, 6 HomoB. Subsequent analyses were performed only on PBMCs and MDMs isolated from HomoB and heteroAB donors to exclude confounding results. Indeed, HomoA individuals express negligible levels of ERAP2/Iso2 and Iso3.

Calu3 cell lines were heterozygous for rs2248374 ERAP2 genotype.

### 3.2. mRNA Expression of ERAPs in PBMCs from Subjects Carrying Different ERAP2 Genotypes Following Microbial Stimulation

**Figure 2:**
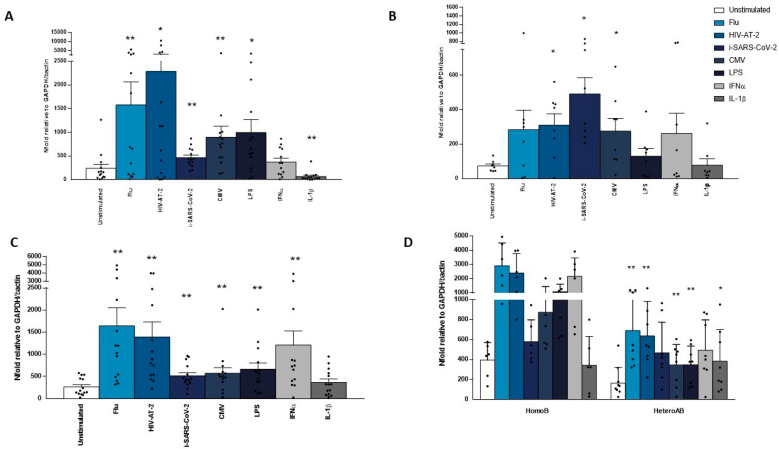
ERAP1, ERAP2/Iso1 and ERAP2/Iso3 mrna expression is increased following microbial stimulation. pbmcs isolated from 8 heteroab and 6 homob individuals were in vitro stimulated with microbial antigens (flu, CMV) inactivated viruses (i-SARS-CoV-2, HIV-AT-2) bacterial by-products (LPS) or inflammatory stimuli (IFNα, IL-1β) for ten h. mRNA expression for ERAP2/Iso3 (**A**), ERAP2/Iso1 (**B**) and ERAP1 (**C**) were assessed by RT-Real-Time PCR. Results are shown as the media of the relative expression units to the glyceraldehyde-3-phosphate dehydrogenase (GAPDH) and β-actin reference genes calculated by the 2^−ΔΔCt^ equation. (**D**) The microbial-dependent genetic control of ERAP2/Iso3 expression is underlined by the observation that its abundances are nearly doubled in HapB homozygotes compared to heterozygotes. Results are expressed as mean ± ES. * = *p* < 0.05; ** = *p* < 0.01.

To verify whether the expression of ERAP2/Iso3 isoform is exclusively flu-specific or it may be triggered by other stimuli, we analyzed its expression on PBMCs isolated from HomoB and HeteroAB HC following HIV-AT-2, i-SARS-CoV-2, CMV, LPS, flu, IFNα, and IL-1β. As expected, the expression of the newly identified ERAP2/Iso3 was significantly augmented in PBMCs from all the subjects included in the study in response to flu (*p* < 0.002). However, even following CMV (*p* < 0.004), LPS (*p* < 0.002), HIV-AT-2 (*p* < 0.003), i-SARS-CoV-2 (*p* < 0.002) and IFNα (*p* < 0.003) stimulation, we observed a significant increase of its expression (Figure 2A). Conversely, IL-1β addition to cell culture did not result in ERAP2/Iso3 induction (Figure 2A). ERAP2/Iso1 expression was observed only in PBMCs from HeteroAB subjects and was induced following all the microbial-stimuli employed plus IFNα but not in response to IL-1β (Figure 2B). However, statistical significance was observed exclusively following HIV-AT-2 (*p* < 0.025), i-SARS-CoV-2 (*p* < 0.03) and CMV (*p* < 0.02). Likewise, ERAP1 expression was induced by all the stimuli employed excepting IL-1β and reached statistical significance following flu (*p* < 0.002), HIV-AT-2 (*p* < 0.03) CMV (*p* < 0.002), i-SARS-CoV-2 (*p* < 0.008) LPS (*p* < 0.05). We also observed a reduction following IL-1β (*p* < 0,001) stimulations (Figure 2C). 

Notably, the microbial-dependent genetic control of ERAP2 isoform usage is sustained by further evidence. Indeed, there was a significant correlation between whole microbial transcript quantity and ERAP2/Iso3 transcript abundances in heterozygotes compared to HapB homozygotes (Figure 2D).

### 3.3. Gene Expression of Immune Selected Effectors in PBMCs Following Microbial Stimulation

**Figure 3:**
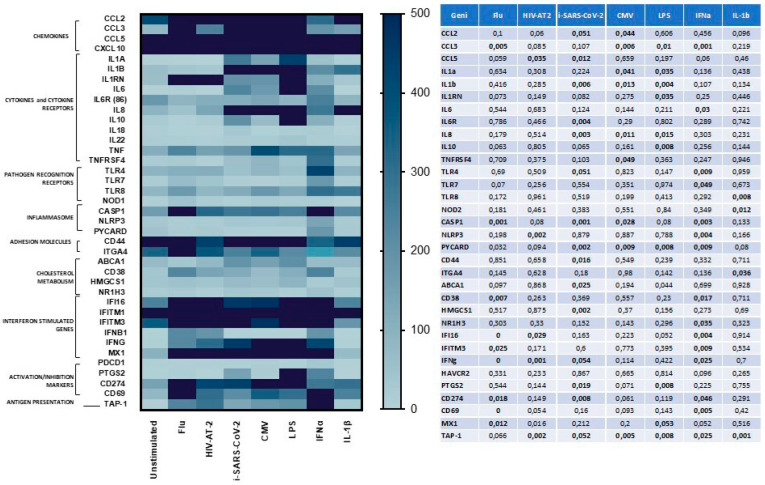
mRNA expression of genes involved in the anti-microbial immune response was modulated in response to different pathogens. Quantigene Plex Gene expression technology was applied to quantify gene expression on PBMCs isolated from 8 HeteroAB and 6 HomoB individuals and stimulated with microbial antigens (flu, CMV) inactivated viruses (i-SARS-CoV-2, HIV-AT-2) bacterial by-products (LPS) or inflammatory stimuli (IFNα, IL-1β). Gene expression (mean values) is shown as a color scale from white to blue (Heatmap). Only statistically significant p values from T-test comparison between unstimulated and stimulated PBMCs are shown in table.

To verify if the increased expression of ERAPs in response to microbial stimulation could be extended to other factors involved in the orchestration of the immune response, mRNA expression of 70 selected effectors was investigated by the innovative Quantigene Plex Gene expression technology. The genes whose mRNA expression was upregulated are implicated in almost all phases of immune response, including: chemokines, cytokines and cytokine receptors, pathogen recognition receptor, inflammasome, cholesterol metabolism, interferon stimulated genes, adhesion molecules, activation/inhibition markers, antigen presentation factors (Figure 3). Notably, the gene expression pattern was partially shared by all the stimuli employed and partially pathogen-specific as summarized in Figure 3. In particular, following i-SARS-CoV-2-stimulation a significantly higher transcription rate was observed for: CCL2 (*p* < 0.05); CCL5 (*p* < 0.035), HMGCS1 (*p* < 0.002), PYCARD (*p* < 0.002), CASP1 (*p* < 0.001), CD44 (*p* < 0.016), CD274 (*p* > 0.008), IL-8 (*p* < 0.003); IL-1β (*p* < 0.006), ABCA1 (*p* < 0.02), IL-6R (*p* < 0.004), CCL3 (*p* < 0.01), IFNγ (*p* < 0.05); TAP1 (*p* < 0.05).

Unlike ERAP2 expression, no differences were observed in mRNA expression levels of all the analyzed genes in PBMCs from HomoB and HeteroAB subjects in response to all the stimuli engaged (data not shown).

### 3.4. mRNA Expression of ERAPs in In Vitro SARS-CoV-2 Infected Calu3 Cell Lines and HIV-Infected PBMCs

**Figure 4:**
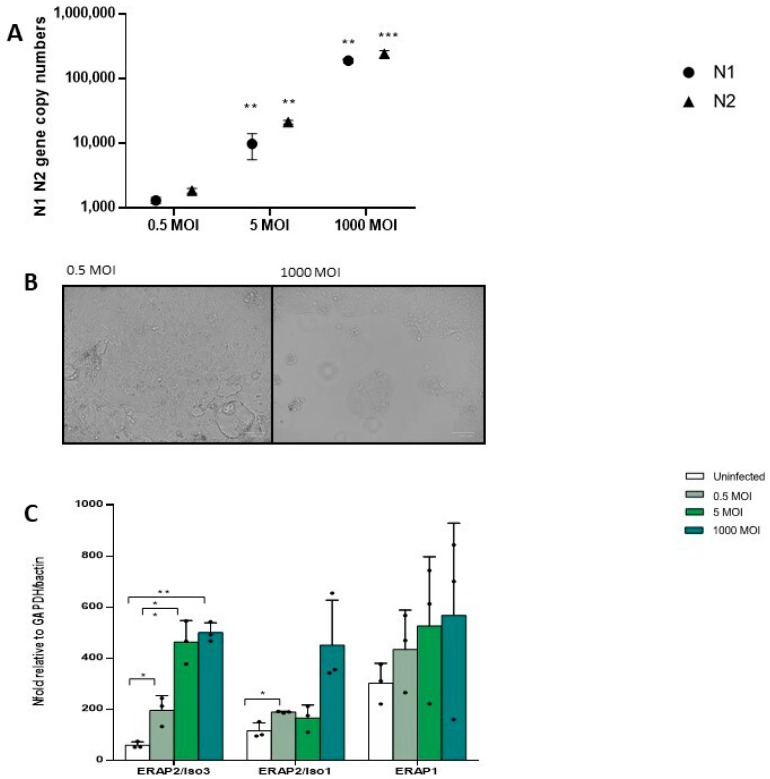
In vitro SARS-CoV-2 infection assay on Calu3 cells. (**A**) SARS-CoV-2 replication was assessed on Calu3 cells infected at 0.5, 5, and 1000 MOI 48 h post-infection. Viral copy number quantification was performed by generating a standard curve from the quantified 2019-nCoV_N positive plasmid control for Nucleocapsid (N) region 1 and 2, showing a significant increase in response to the increased viral input. (**B**) SARS-CoV-2-induced cytopathic effects were assessed in Calu3 infected cells. Representative images of SARS-CoV-2 infected-cells at 0.5 and 1000 MOI are reported. At 48 h post-infection, typical cytopathic effects, including cell rounding, detachment, degeneration, and syncytium formation were seen only in cells infected at 1000 MOI. Cells were imaged by optical microscope observation (ZOE™ Fluorescent Cell Imager, Bio-Rad, Hercules, CA, USA). (**C**) ERAP2/Iso3, Iso1, and ERAP1 mRNA expression by in vitro SARS-CoV-2 infected Calu3 cells increased according to the rising viral input. Mean values ± ES are reported. * = *p* < 0.05; ** = *p* < 0.01; *** = *p* < 0.001. MOI = multiplicity of infection.

To verify whether ERAP2/Iso3 expression varies in response to growing viral concentrations we adopted two in vitro model of infection. Thus, Calu3 cell lines were infected with different SARS-CoV-2 viral input and after 48 hours’ viral replication as well as ERAP mRNA expression were assessed. As expected, SARS-CoV-2 replication increased according to the rising viral input as assessed analyzing both N1 (MOI 0.5 vs. 5: *p* < 0.01; MOI 0.5 vs. 1000: *p* < 0.001) and N2 (MOI 0.5 vs. 5: *p* < 0.01; MOI 0.5 vs. 1000: *p* < 0.001) (Figure 4A). Images of cellular cytopathic effect on SARS-CoV-2 infected cells at 48 h showed that despite robust SARS-CoV-2 replication in Calu3 cells, substantial cell death was detected only in cells infected with 1000 MOI (Figure 4B). Notably, this increase was coupled with a progressive rise of ERAP2/Iso3 expression compared to the uninfected condition (MOI 0.5: *p* < 0.04; MOI 5: *p* < 0.01; MOI 1000: *p* < 0.01). Likewise, ERAP2/Iso1 and ERAP1 expression was induced in a viral-dose dependent manner, although statistical significance was observed only for ERAP2/Iso1 (MOI 0.5: *p* < 0.05) (Figure 4C). 

**Figure 5:**
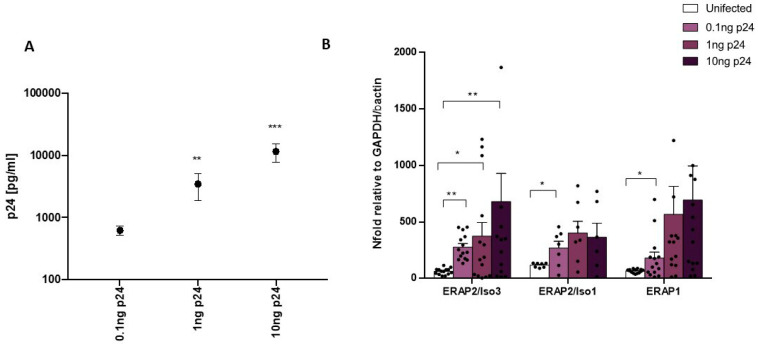
In vitro HIV-1 infection assay on PBMCs. (**A**) HIV-1 replication was assessed by p24 quantification on PBMCs isolated from 8 HeteroAB and 6 HomoB individuals infected with 0.1, 1, and 10 ng p24 HIV-1Bal/1 × 10^6^ PBMCs 5 days post infection. Results showed a significant increase in viral replication according to the increased viral input. (**B**) ERAP2/Iso3, Iso1, and ERAP1 mRNA expression by in vitro HIV-1-infected PBMCs increased according to the rising viral input. Mean values ± ES are reported. * = *p* < 0.05; ** = *p* < 0.01; *** = *p* < 0.001.

In vitro HIV-1 infection of PBMCs isolated from HapB HC produced similar results. Indeed, as the viral input raised, viral replication quantified through p24 concentration analyses at 5 days post infection increased (0.1 vs. 1 ng p24 HIV-1_Bal_/1 × 10^6^ PBMCs: *p* < 0.02; 0.1 vs. 10 ng p24 HIV-1_Bal_/1 × 10^6^ PBMCs: *p* < 0.001) (Figure 5A). Likewise, ERAP2/Iso3 gene expression increased compared to the uninfected condition (0.1 ng p24 HIV-1_Bal_/1 × 10^6^ PBMCs: *p* < 0.003; 0.1 ng p24 HIV-1_Bal_/1 × 10^6^ PBMCs: *p* < 0.04; 10 ng p24 HIV-1_Bal_/1 × 10^6^ PBMCs: *p* < 0.03) (Figure 5B). ERAP2/Iso1 (0.1 ng p24 HIV-1_Bal_/1 × 10^6^ PBMCs: *p* < 0.05) and ERAP1 (0.1 ng p24 HIV-1_Bal_/1 × 10^6^ PBMCs: *p* < 0.05) mRNA levels showed a similar trend (Figure 5B).

### 3.5. ERAP2/Iso3 Protein Production by MDMs from HeteroAB Subjects Following Microbial Specific Stimulation

**Figure 6:**
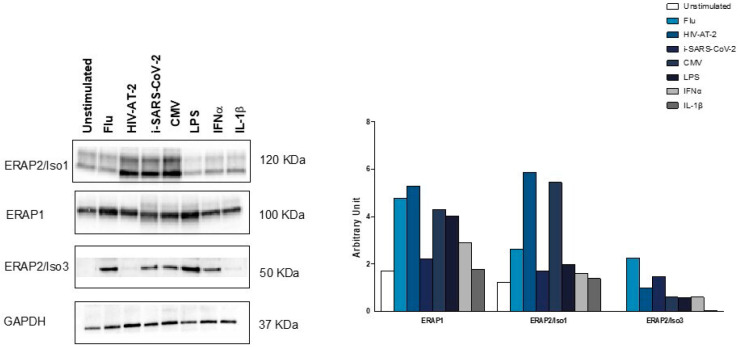
ERAP1, ERAP2/Iso1, and ERAP2/Iso3 production in pathogen stimulated monocyte-derived macrophages (MDMs). MDMs differentiated from 3 HeteroAB participants stimulated for 36 h with microbial antigens (flu, CMV) inactivated viruses (i-SARS-CoV-2, HIV-AT-2) bacterial by-products (LPS) or inflammatory stimuli (IFNα, IL-1β) were tested for protein using primary antibodies specific to a ERAP1 (goat polyclonal), ERAP2 and β-actin. Proteins extracted from the 3 HeteroAB subjects were sub-pooled for WB analyses. Histograms representing ERAP1, ERAP2/Iso1, and ERAP2/Iso3 densitometric quantification. Quantification was performed by Quantity One 4.6.6 software (Bio-Rad) and normalization was permed on GAPDH.

To verify if the short ERAP2/Iso3 isoform would function as an RNA or is translated into a protein product we performed a western blot assay on MDMs from 3 HeteroAB subjects triggered with different microbial antigens. Remarkably, the antibody which recognizes the full-length ERAP2 (Iso1) was able to detect also one short protein isoform (∼50 kDa) in CMV, flu, HIV-AT-2, i-SARS-CoV-2, LPS, IFNα stimulated cells from HeteroAB subjects, suggesting the translation of the short microbial-specific ERAP2/Iso3 (Figure 6). Conversely, following IL-1β stimulation, only ERAP2/Iso1 isoform was detected. The production of ERAP1 protein was observed in all the stimulated conditions except IL-1β (Figure 6). As the proteins extracted from the 3 HeteroAB subjects were sub-pooled for WB analyses, statistical evaluation of the results was not possible.

## 4. Discussion

Given the documented role of ERAP2 in antigen presentation [20] and viral infections [21], we examined the genetic control of ERAP2 transcripts in the human antimicrobial response. In particular, based on the results recently reported by Ye and co-workers [19], we investigated if the expression of the recently characterized ERAP2/Iso3 is flu-specific or if it can be triggered by other microbial stimuli. Our results suggest that: (1) ERAP2/Iso3 mRNA expression is not restricted to flu-infection but it can be prompted by other pathogens including HIV, SARS-CoV-2, CMV, Bacteria (LPS); (2) ERAP2/Iso3 mRNA is translated into a protein following microbial induction; (3) ERAP2/Iso3 mRNA expression is sensitive to viral concentration. 

Remarkably, Ye et al. did not detect ERAP2/Iso3 expression in IFNB1 stimulated cells leading to the conclusion that the transcription of novel ERAP2 isoforms is likely initiated by viral sensing pathways upstream of type 1 interferon signaling [19]. Conversely, in our cell culture condition, IFNα-stimulation was able to induce ERAP2/Iso3 expression in a consistent way, suggesting the participation of type 1 interferon cascade to the induction of ERAP2/Iso3 transcription, as already documented for the wild type forms of ERAP1 and 2 [26]. The different cell kinds and IFN subtypes adopted in the two experimental settings could at least partially justify the discrepancies reported in the two studies, but further analyses are needed to clarify the pathways and molecules induced by microbial exposure, directly responsible for ERAP2/Iso3 synthesis. For example, in our study IL-1β stimulus was not able to trigger the expression of any ERAP variants, further strengthening the assumption of a specific response of ERAP gene transcription controlled by pathogen exposure. Furthermore, as IFNα is also strongly associated to various type of I IFN conditions such as Sjogren’s disease [27], systemic lupus erythematosus [28] and Scleroderma [29] it would be valuable to verify if ERAP2/Iso3 expression varies in patients affected by these pathologies and/or following administration of IFNα therapy.

The discovery of these new ERAP2 isoforms is of great importance and gives a plausible explanation to the maintenance of one of the major gene expression quantitative trait loci (eQTL) and alternate isoform usage in most tissues and cell types [19,30]. Indeed, until last year, the preservation of HapB at intermediate frequency in human population was almost unexplained, as its transcript was believed to be addressed to NMD [9,10]. The identification of this previously uncharacterized short isoform, ERAP2/Iso3, transcribed from HapB, results in the partial rescue of ERAP2 expression, suggesting its involvement in the anti-microbial response. 

The mechanism of action and functional impact of this new genetic variant to host defense; however, is still indefinite. The lack of the aminopeptidase domain proves that it does not directly participate in the shaping of antigenic peptides to be presented to CD8+ T cells. However, in 2005 Saveanu et al. conducted an immunoblot analysis detecting the presence of ERAP1-ERAP2 heterodimers and possibly homodimers of each enzyme [4]. This crystallographic dimer is described as mediated by domains I and II of the enzyme that is missing from ERAP2/Iso3 [31]. However, this observation has been reiterated by Ye et al. who hypothesized that ERAP2 isoforms could have a dominant-negative effect on either ERAP1 and possibly ERAP2 wt, through the formation of hetero- or homodimeric complexes [15]. Despite still speculative the fact that ERAP2/Iso3 may exert such an effect cannot be ruled out: this, in turn, could lead to an altered peptide processing, which could confer an advantage/disadvantage against infections by presenting a more/less immunogenic antigen repertoire. In relation to this aspect, previous studies have demonstrated that the functional skills of ERAP monomers, homo, or heterodimers may significantly differ in terms of both substrate specificity and trimming efficiency [32]. In particular, ERAP1/2 dimerization creates complexes with superior peptide-trimming efficacy and a higher affinity towards ERAP1 preferential substrates. This is allowed by the adoption of a modified physical conformation by ERAP1, caused by its interaction with ERAP2, which mainly works as an enhancer of ERAP1 role upon dimer assembling [33]. Further studies are needed to verify if also ERAP2/Iso3 physical interaction with the wt ERAP variants prompts an allosteric effect able to modify basic enzymatic parameters and to improve their substrate-binding affinity. However, the observation that ERAP2/Iso3 mRNA is translated into a protein allows to speculate on the generation of a new ERAP member which further contributes to enrich the non-redundant, yet a complete and potent system of aminopeptidases, warranting an efficient trimming of various kinds of precursors. 

The results obtained in this study definitely establish a link between invading pathogens and ERAP2/Iso3 expression which further strengthens the significance of the results reported by Ye and collaborators. Supporting this hypothesis, we observed that ERAP2/Iso3 expression progressively increased in response to growing doses of viral input in both SARS-CoV-2 and HIV-1 in vitro infection assay, as if the production of this genetic variant, as well as one of the other elements within the ERAP family, were directly dependent on the viral dose of exposure. Additionally, the observation that the increased expression of ERAP2/Iso3 in response to pathogen exposure is accompanied by the modulation of many other determinants (chemokines, cytokines, pathogen recognition receptor, inflammasome, interferon-stimulated genes, adhesion molecules, activation/inhibition markers, antigen presentation elements) orchestrating the anti-microbial immune response, further supports its direct intervention in this defensive pathway. Notably, as its expression is increased by a wide range of microbial stimuli including viral antigens, inactivated virus as well as bacterial by-products, it is possible to assume that ERAP2/Iso3 expression is not pathogen-specific, but it’s secondary to the activation of an antimicrobial cascade commonly shared by different pathogens. Meanwhile, we cannot exclude that ERAPs responses observed following microbial stimulations and in vitro viral infections result from an erroneous transcription and translation due to pathogen-induced cellular stress. Indeed, as ERAP1 and ERAP2 expression has been demonstrated to be prompted by IFNγ stimulation, ERAPs production could be secondary to the innate immune response of the cells to infections, rather than to an immune response or an immune evasion mechanism. Further analyses will be necessary to verify this hypothesis.

The involvement of ERAPs in modulating viral infections is widely recognized as recently reviewed in [34]. Several studies, indeed, have demonstrated the intervention of ERAP genetic variants in the life cycle of HCV, flu, CMV, HPV, HIV, and other pathogens at different levels. In particular, studies performed by our research group have established an association between ERAP2/Iso1 and HIV-infection in terms of both susceptibility [17,35] and progression [36]. However, to our knowledge ERAP expression and/or genetic variants have been correlated to the recent coronavirus disease 2019 (COVID-19), provoked by SARS-CoV-2, only by two recent studies [34,37,38]. In the first one by Stamatakis et al. ERAP2 trimming ability has been investigated in SARS-CoV-2 infection together with ERAP1 and IRAP, and it has been proved as the most stable of the enzymes generating optimal length antigenic peptides for HLA binding [38]. In the second one by Lu et al. by examining 193 deaths from 1,412 confirmed infections in a group of 5,871 UK Biobank participants tested for the virus, rs150892504 variant in ERAP2 gene came up as potentially being implicated in risk from SARS-CoV-2 infection. Although rs150892504 variant is not in linkage disequilibrium with rs2248374, this finding suggests the involvement of ERAP2 in the modulation of SARS-CoV-2 infection. Such an assumption is further supported by other intriguing observations. This virus enters the cells by spike protein binding to ACE2 (angiotensin-converting enzyme 2), which is responsible for the conversion of angiotensin I to angiotensin I-9 and of angiotensin II to angiotensin I-7, an effective vasodilator, thus working as a negative regulator of the renin-angiotensin system (RAS) Our results, for the first time demonstrate that SARS-CoV-2 exposure triggers the expression of ERAP1, ERAP2/Iso1 and also the recently detected ERAP2/Iso3 in a dose-dependent mode, suggesting its participation in the control of the anti-SARS-CoV-2 response. This observation is far more important, considering that besides their involvement in the antigen presentation pathway, ERAPs display several key anti-SARS-CoV-2 functions. Indeed, they intervene in the RAS, where ERAP1 efficiently cleaves angiotensin II to angiotensin III and IV, and ERAP2 cuts angiotensin III to angiotensin IV, thus influencing both ACE2 virus receptor bio-availability and blood pressure levels [39]. Furthermore, ERAPs modulate the proteolytic cleavage of IL-6 receptor (IL-6Rα) [40] a function which can improve the clinical conditions in COVID-19 patients, as recently documented following its pharmacological inhibition by Tocilizumab [41]. Last but not least, Ranjit and colleagues recently demonstrated that sex-specific differences in ERAP1 modulation influence blood pressure and RAS responses [42], and a male bias in mortality has emerged in the COVID-19 pandemic since the very beginning. As ERAPs genetic variants have been demonstrated to orchestrate and condition the result of infection of coronavirus in other animal species [43,44] detailed studies investigating SARS-CoV-2-host interplay is absolutely mandatory.

Another query which needs to be addressed in the near future concerns the cellular localization of ERAP2/Iso3 isoform. In particular, it would be interesting to verify if, as with the wt ERAP variants [17], even ERAP2/Iso3 may be secreted into the extracellular milieu following inflammatory stimulation, with which substrates it may interact, which functions may eventually exert in this environment and if its administration can interfere with viral replication.

Considering the key role played by ERAPs in antigen processing and presentation, it is plausible that these aminopeptidases may be potential targets and controllers of the pathogenicity of infectious diseases, shaping the susceptibility and response to microbial infections. The recent acquisition of ERAP intervention even in the modulation of innate immunity further reinforces this assumption.

Given the growing number of viral epidemics, the identification of molecular mechanisms driven by factors such as ERAPs that can interfere, control or modulate viral replication is unequivocally needed as they could be widely exploited for the inception of future, still unknown viral infections.
